# Luteolin Attenuates Hypertension via Inhibiting NF-κB-Mediated Inflammation and PI3K/Akt Signaling Pathway in the Hypothalamic Paraventricular Nucleus

**DOI:** 10.3390/nu15030502

**Published:** 2023-01-18

**Authors:** Hong-Li Gao, Xiao-Jing Yu, Yu-Qi Feng, Yu Yang, Han-Bo Hu, Yu-Yang Zhao, Jia-Hao Zhang, Kai-Li Liu, Yan Zhang, Li-Yan Fu, Ying Li, Jie Qi, Jin-An Qiao, Yu-Ming Kang

**Affiliations:** 1 Key Laboratory of Environment and Genes Related to Diseases of Education Ministry of China, Department of Physiology and Pathophysiology, Shaanxi Engineering and Research Center of Vaccine, Xi’an Jiaotong University School of Basic Medical Sciences, Xi’an 710061, China; 2 Institute of Pediatric Diseases, Xi’an Children’s Hospital, Xi’an 710003, China

**Keywords:** luteolin, hypothalamic paraventricular nucleus, PI3K/Akt/NF-κB signaling pathway, infammatory cytokines, oxidative stress, hypertension

## Abstract

Background: Luteolin is widely distributed among a number of vegetal species worldwide. The pharmacological effects of luteolin are diverse and amongst antioxidant, free radical scavenging, and anti-inflammatory activities. Preliminary study showed that luteolin can ameliorate hypertension. However, the precise mechanism needs further investigation. There is no evidence that luteolin affects the paraventricular nucleus of the hypothalamus (PVN), a brain nucleus associated with a critical neural regulator of blood pressure. Our main aim was to explore the effect of luteolin on the PI3K/Akt/NF-κB signaling pathway within the PVN of hypertensive rats. Methods: spontaneously hypertensive rats (SHRs) and corresponding normotensive control rats, the Wistar Kyoto (WKY) rats were divided into four groups and subsequently treated for 4 weeks with bilateral PVN injections of either luteolin (20 µg/0.11 µL, volume: 0.11 µL/h) or vehicle (artificial cerebrospinal fluid). Results: luteolin infusion to the PVN significantly decreased some hemodynamic parameters including the mean arterial pressure (MAP), heart rate (HR), circulating plasma norepinephrine (NE) and epinephrine (EPI). Additionally, there was a decrease in the expressions of the phosphatidylinositol 3-kinase (p-PI3K) and phosphorylated protein kinase-B (p-AKT), levels of reactive oxygen species (ROS), NAD(P)H oxidase subunit (NOX2, NOX4) in the PVN of SHRs. Meanwhile, the expression of inflammatory cytokines and the activity of nuclear factor κB (NF-κB) p65 in the PVN of SHRs were lowered. Furthermore, immunofluorescence results showed that injection of luteolin in the PVN reduced the expression of tyrosine hydroxylase (TH), and increased that of superoxide dismutase (SOD1) and the 67-kDa isoform of glutamate decarboxylase (GAD67) in the PVN of SHRs. Conclusion: Our novel findings revealed that luteolin lowered hypertension via inhibiting NF-κB-mediated inflammation and PI3K/Akt signaling pathway in the PVN.

## 1. Introduction

Hypertension, as characterized by elevated blood pressure (140/90 mmHg), is a leading powerful risk factor for cardiovascular diseases [1,2]. Unfortunately, the cure rate of hypertension in the world is still unsatisfactory. Once diagnosed with hypertension, the patient will take medicine for life [3]. The basic reason is that the pathogenesis of this condition is not clearly elucidated. In recent years, more and more evidence support the crucial role played by the paraventricular nucleus of the hypothalamus (PVN) is important in the onset and progression of high blood pressure [4,5]. The PVN is a small bilateral structure located bilaterally around the third ventricle [5], which plays an important modulatory role in sympathetic and cardiovascular systems [6,7]. In hypertension, the hyperactivation of the sympathetic nerve is significantly principal to the changes of molecular signals within PVN.

In the PVN, neuroinflammation, and generation of reactive oxygen species (ROS) increase sympathetic activity in hypertension [8]. Neuroinflammation as estimated by proinflammatory cytokine level, activation nuclear factor κB (NF-κB), and ROS due to the intensification of oxidative stress reaction were higher and superoxide dismutase (SOD) expression within PVN was lower in various models of hypertensive rats [9,10]. Studies have shown that NF-κB is implicated in the regulation of a variety of inflammatory cell responses, and has been used for the targeted treatment of many inflammatory diseases. Research shows that hypertension is a chronic inflammatory reaction disease [11]. Blocking the NF-κB pathway in PVN can improve hypertension by lowering peripheral sympathetic nerve activity toward peripheral organs [12]. The PVN level of Phosphatidylinositol 3-kinase (PI3K) and the phosphorylated protein kinase-B (AKT) was increased in spontaneously hypertensive rats (SHRs) [13]. The up-regulated PI3K/Akt signaling pathway contributes to sympathetic overdrive and hypertension [14]. It has already been found blockade of the PI3K/Akt signal pathway effectively improves hypertension [15].

**Figure 1:**
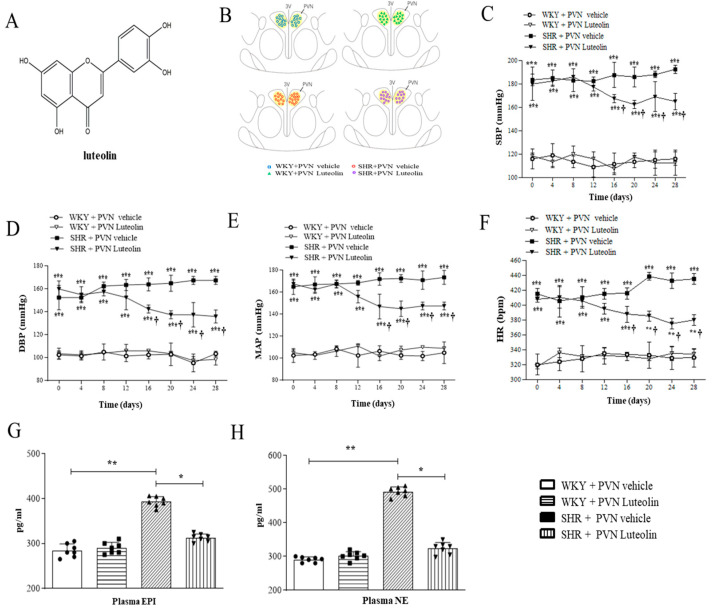
Effects of PVN luteolin on blood pressure, the plasma levels of NE, and EPI. (**A**) Structural formula of luteolin. (**B**) Schematic representation of microinjection sites. (**C**) The systolic blood pressure (SBP) in different groups. (**D**) The diastolic blood pressure (DBP) in different groups. (**E**) The mean arterial pressure (MAP) in different groups. (**F**) Heart rate (HR) in different groups. (**G**) ELISA detection of the plasma level of EPI in different groups. (**H**) ELISA detection of plasma level NE in different groups. Values are mean ± SEM. * *p* < 0.05, ** *p* < 0.01 and *** *p* < 0.001 versus WKY groups (WKY + PVN vehicle or WKY + PVN luteolin); † *p* < 0.05 SHR + PVN vehicle versus SHR + PVN luteolin.

Luteolin (3′,4′,5,7-tetrahydroxyflavone, Figure 1A) is widely distributed among the number of vegetal species worldwide. It was originally isolated from the leaves, stems, and branches of the mignonette. At present, it is mainly found in chrysanthemum, Prunella vulgaris, artichoke, perilla, celery, groundnut, and other natural Chinese medicinal materials and vegetable and fruits. Luteolin, mostly in the form of glycosides, exists in a variety of plants. These plants have a high content of whole-leaf blue orchid, pepper, wild chrysanthemum, honeysuckle, and perilla [16,17]. In previous studies, we also found that some flavonoids such as apigenin, which can reduce elevated blood pressure in SHRs [18], and proanthocyanidins can inhibit the progression of renovascular hypertension [19]. Accumulating evidence has suggested that luteolin not only abates inflammation and oxidation, and reduce the damage caused by inflammatory factors and excessive reactive oxygen species, but also protects normal tissues and cells, protects the nervous system, and reduces the occurrence of neuropathy [20]. The pharmacological effects of luteolin are diverse and amongst antioxidant, free radical scavenging, and anti-inflammatory activities. In addition, this molecule has been proven to have a brain-protective effect in many neurodegenerative conditions, including Alzheimer’s disease, Parkinson’s disease and cerebral ischemia [21]. Luteolin has rich pharmacological effects and strong medicinal value. It has a definite curative effect against major diseases such as COVID-19/asthma comorbidity [22], skin cancer [23], prostate cancer [24], and atopic dermatitis [25] and is of great significance to human health. The benefits of luteolin are protecting against cardiovascular disease, such as hypertension [26,27]. However, to date, the underlying central molecular mechanism by which luteolin improves hypertension remains elusive.

Therefore, this study was designed to identify whether luteolin could ameliorate sympathetic activation and hypertension in SHRs, and whether NF-κB-mediated inflammation and PI3K/Akt signaling pathway within PVN participate in the central molecular anti-hypertensive mechanisms of luteolin.

## 2. Materials and Methods

### 2.1. Animal

For the purpose of this study, healthy adult male Wistar Kyoto (WKY) rats and spontaneously hypertensive rats (SHRs), of twelve weeks were provided by Vital River Biological Co., Ltd., Beijing, China. This study passed the ethical clearance of the Institutional research ethics committee (No.XJTULAC2020-63). All procedures complied with the Guide for the Care and Use of Laboratory Animals (NIH publication, 8th edition, 2011).

### 2.2. Methods of Administration to PVN

To ensure that the luteolin works through the PVN, we used the method of chronic administration of luteolin to bilateral PVN, as described previously [28,29].

### 2.3. Animal Treatments

SHRs and WKY rats were randomly assigned to the following groups. Group I: WKY + PVN vehicle; Group II: WKY + PVN luteolin; Group III: SHRs + PVN vehicle; Group IV: SHRs + PVN luteolin; luteolin (>98% purity) was obtained from Sigma-Aldrich (Saint Louis, MO, USA). Vehicle (artificial cerebrospinal fluid, aCSF) or luteolin (20 µg/0.11 µL, volume: 0.11 µL/h) was injected into the bilateral PVN of rats for 28 days [10,19]. Blood and PVN tissue of all rats were collected for subsequent molecular experiments.

### 2.4. Measurement of SBP, DBP and MAP

Cardiovascular parameters, including blood pressure and heart rate (HR) changes, were recorded from each rat via the noninvasive method of tail-cuff occlusion as previously described [30].

### 2.5. Immunofluorescence Staining

Immunofluorescence studies were performed to assess the level of SOD-1, NOX2, IL-1β, p-PIKKβ, tyrosine hydroxylase (TH) and the 67-kDa isoform of glutamate decarboxylase (GAD67) in the PVN. The primary antibodies were SOD-1 (1:200 dilution, ab-16831, Abcam, Cambridge, UK), NOX2 (1:500 dilution, ab-31092, Abcam, Cambridge, UK), TH (1:50 dilution, AB152, Millipore Sigma, Temecula, CA, USA), and GAD67 (1:100 dilution, ab-26116, Abcam, Cambridge, UK). IL-1β (1:200 dilution, ab-9722, Abcam, Cambridge, UK) and p-PIKKβ (1:50 dilution, sc-21661, Santa Cruz, CA, USA). The specific method is the same as that described previously [10].

### 2.6. Dihydroethidium Staining

To evaluate ROS generation in PVN, we chose to use Dihydroethidium (DHE) staining. This staining uses the DHE (Molecular Probes, Eugene, OR, USA) and was conducted following the previously described method [31].

### 2.7. Real-Time Quantitative PCR

The NOX4, interleukin 6 (IL-6) and interleukin 1β (IL-1β) gene expression were quantified through real-time PCR assays. The specific operation process is the same as previously described [32,33] and the following primer sequences were used (See Table 1).

### 2.8. Biochemical Assays

Circulating norepinephrine (NE) and Epinephrine (EPI) were quantified using the commercially available ELISA kits. Procedures are conducted following the manufacturer’s guidelines.

### 2.9. Western Blotting

The method has been described previously [34]. PI3K, phosphorylated PI3K, Akt, and phosphorylated Akt in the PVN were measured with Western blotting. Antibody of PI3K (1:1000, #4257S, Cell Signaling, Danvers, MA, USA), phosphorylated PI3K (1:2000, #4228S, Cell Signaling, Danvers, MA, USA), Akt (1:1000, #4685S, Cell Signaling, Danvers, MA, USA), phosphorylated Akt (1:2000, #4060S, Cell Signaling, Danvers, MA, USA) were used. β-actin was developed as a reference to normalize the data of the WKY + PVN vehicle group. 

### 2.10. Statistical Analysis

Repeated-measures ANOVA was used to analyze data related to SBP, DBP, and MAP. As for other data, two-way ANOVA with Turkey’s multiple comparison tests was employed. All statistical analyses were conducted using GraphPad Prism 8.0 Software for windows (GraphPad Software, La Jolla, CA, USA). *p* < 0.05 was considered to be statistically significant and results were expressed as mean ± SEM.

## 3. Results

### 3.1. Luteolin Improved Elevated Blood Pressure and Heart Rate in SHRs

Figure 1A shows the structural formula of luteolin, Figure 1B is the schematic reconstruction of microinjection sites in PVN. luteolin lowered systolic blood pressure (SBP) to 160 ± 7 mmHg (Figure 1C); Luteolin lowered diastolic blood pressure (DBP) to 137 ± 5 mmHg (Figure 1D); Luteolin lowered the mean arterial pressure to 152 ± 3 mmHg (Figure 1E); Luteolin lowered heart rate (HR) to 380 ± 8 beats/min (Figure 1F) without affecting those parameters in WKY rats.

### 3.2. Luteolin Reduced the Circulating Levels of NE and EPI

SHRs presented increased the circulating levels of EPI (Figure 1G) and NE (Figure 1H) in the blood. While having no effect on both NE and EPI levels in WKY rats. Luteolin significantly lowered the circulating levels of these two catecholamines in SHRs.

### 3.3. Luteolin Alleviated Oxidative Stress in the PVN of SHRs

**Figure 2:**
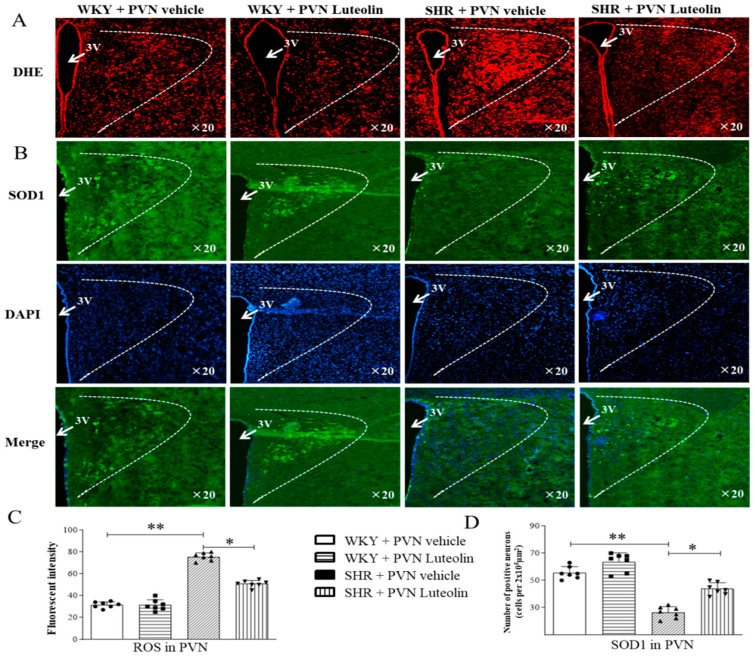
Effects of PVN luteolin on ROS and SOD1 in PVN. (**A**) A representative fluorescent microphotographs depicting the superoxide production as detected with dihydroethidium staining (DHE, red fluorescence). (**B**) A representative fluorescent microphotographs revealing SOD1 positive cells (green fluorescence). Quantification analysis of red fluorescence of DHE (**C**) and green fluorescence of SOD1 (**D**) positive cells in PVN of SHRs and WKY rats, treated with luteolin or aCSF. Values are the mean ± SEM from 7 rats/group. * *p* < 0.05, ** *p* < 0.01. 3V, third ventricle.

Oxidative stress reaction will lead to cell and tissue damage; it can activate a variety of transcription factors, resulting in abnormal expression of some inflammatory pathway-related genes. Increased blood pressure in SHRs was associated with a pro-oxidative status of the PVN. This was materialized by an elevated level of ROS (suggested by fluorescence intensity in DHE staining, Figure 2A,C) in the PVN of SHRs and was reduced by infusion of luteolin, as suggested by the DHE staining. Such increased ROS was reduced by infusion of luteolin.

Immunofluorescence staining showed a lower SOD1 positive cell count (Figure 2B,D) in the PVN of SHRs. However, luteolin infusion in SHRs led to an increase in the number of SOD1-positive cells.

**Figure 3:**
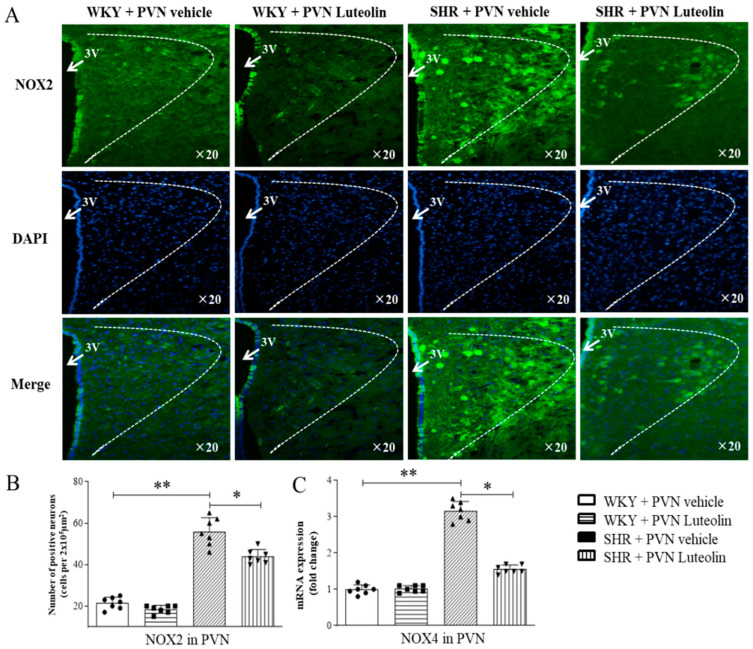
Effects of PVN luteolin on NOX2 and NOX4 in PVN. (**A**) A representative immunomicrographs presenting NOX2 positive cell (green fluorescence). (**B**) Quantification of the number of NOX2 positive cells in PVN of SHRs and WKY rats, with or without luteolin treatment. (**C**) Determination of the expression level of mRNA of NOX4 changes in PVN of SHRs and WKY rats treated with luteolin or aCSF. Values are the mean ± SEM from 7 rats/group. * *p* < 0.05, ** *p* < 0.01. 3V, third ventricle.

An increased number of NOX2 positive cells (Figure 3A,B) and increased mRNA expression levels of NOX4 (Figure 3C) can be found in the paraventricular nucleus of the hypothalamus of SHRs. Interestingly, Luteolin infusion in SHRs reduced of the number of NOX2 positive cells and mRNA levels of NOX4 while restoring the number of SOD1 positive cells. Importantly, Luteolin did not induce any significant change in the expression of oxidative stress markers in the PVN in WKY rats.

### 3.4. Luteolin Reduced the Levels of Inflammatory Cytokines in the PVN of SHRs

**Figure 4:**
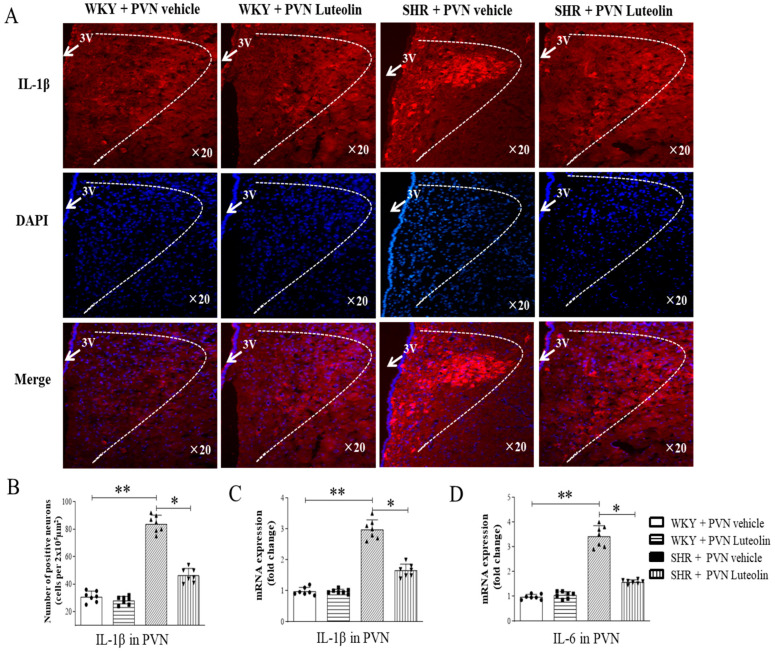
Effects of PVN luteolin on inflammatory cytokine in PVN. (**A**) A representative immunomicrographs presenting IL-1β positive cell (red fluorescence). (**B**) Quantification of the number of IL-1β positive cells in PVN of SHRs and WKY rats, with or without luteolin treatment. (**C**) Determination of the expression level of mRNA of IL-1β and IL-6 (**D**) changes in PVN of SHRs and WKY rats treated with luteolin or aCSF. Values are the mean ± SEM from 7 rats/group. * *p* < 0.05, ** *p* < 0.01. 3V, third ventricle.

Neuroinflammation in the PVN is closely related to the increase of sympathetic drive in hypertensive animal models. We also assessed the level of inflammatory cytokines in animals in response to luteolin treatment. The experimental results of the present study found that there was a significant rise in the number of IL-1β positive cells (Figure 4A,B) and in the mRNA expression level of IL-1β (Figure 4C). Similarly, SHRs had increased the number of IL-6 positive cells (Figure 4D) in the PVN. All these changes were reverted by luteolin infusion.

### 3.5. Luteolin Decreased the Activity of NF-κB in the PVN of SHRs

**Figure 5:**
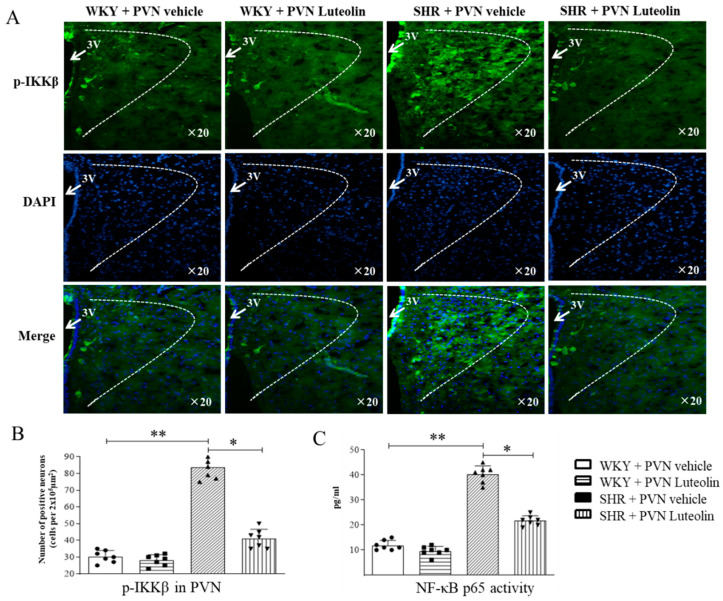
Effects of PVN luteolin on NF-κB in PVN. (**A**) A representative immunofluorescence image showing p-IKKβ positive cell (green fluorescence). (**B**) Quantification of p-IKKβ positive cell count in PVN of SHRs and WKY rats, with or without luteolin treatment. (**C**) Statistical analysis of NF-κB activity in PVN of SHRs and WKY rats treated with luteolin or aCSF. Values are the mean ± SEM from 7 rats/group. * *p* < 0.05, ** *p* < 0.01. 3V, third ventricle.

Triggered NF-κB signal can produce a variety of neurotoxic factors, including many inflammatory cytokines, thus promoting the development of blood pressure. To evaluate the activity of NF-κB in the PVN, immunofluorescence staining and Elisa were used. Results showed an increased number of p-IKKβ positive cells (Figure 5A,B) and elevated activity of the NF-κB p65 (Figure 5C) in the PVN of SHRs. The number of p-IKKβ positive cells and the NF-κB p65 activity were reduced by luteolin infusion in SHRs but not in WKY.

### 3.6. Luteolin Reduced the Production of TH in the PVN of SHRs

**Figure 6:**
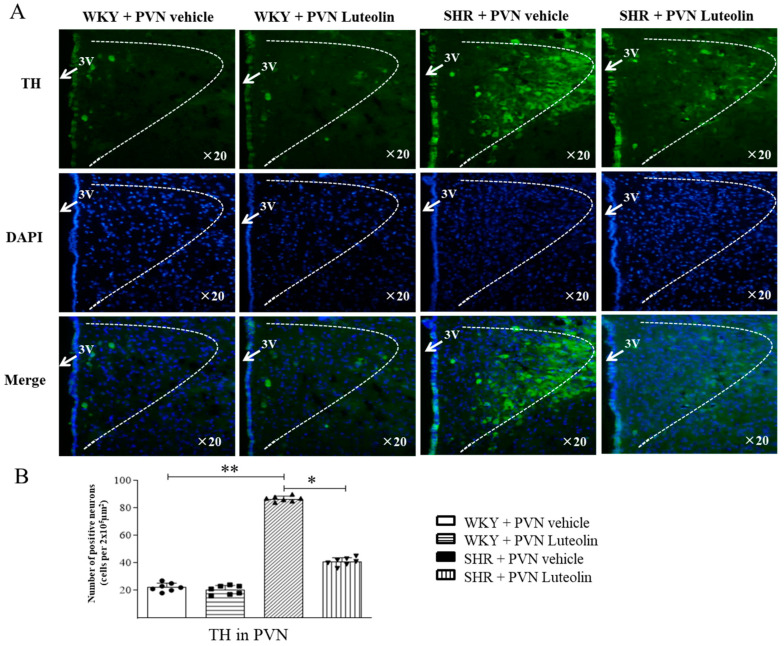
Effects of PVN luteolin on TH in PVN. (**A**) A representative immunomicrographs reporting TH positive cell (green fluorescence). (**B**) Quantification of the number of TH positive cells in PVN of SHRs and WKY rats, with or without luteolin treatment. Values are the mean ± SEM from 7 rats/group. * *p* < 0.05, ** *p* < 0.01. 3V, third ventricle.

Immunofluorescence staining shows that hypertension in SHRs is associated with increased production of TH (Figure 6A,B) in the PVN which was reduced by the chronic infusion of luteolin in SHRs but not in WKY rats.

### 3.7. Luteolin Decreased the Production of GAD67 in the PVN of SHRs

**Figure 7:**
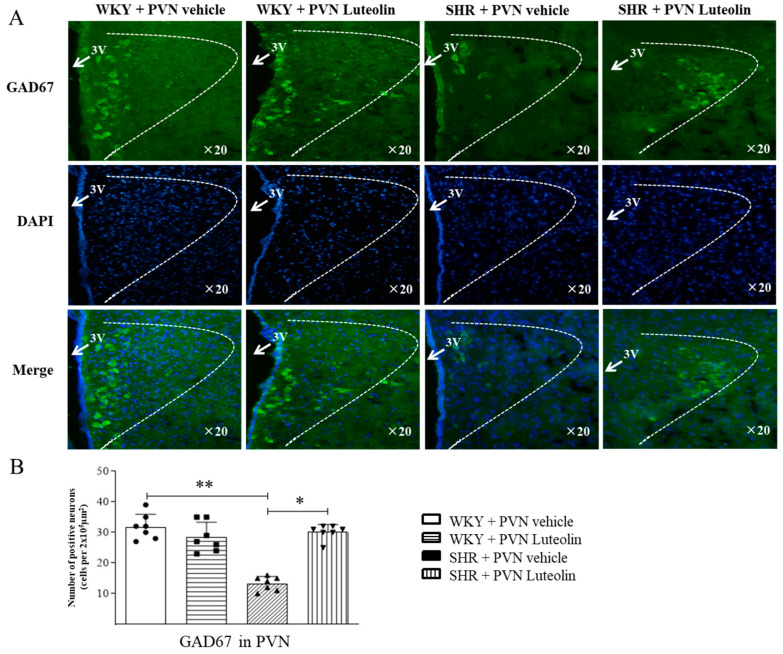
Effects of PVN luteolin on GAD67 in PVN. (**A**) A representative immunomicrographs showing GAD67 positive cell (green fluorescence). (**B**) Statistical quantification of GAD67 positive cells in PVN of SHRs and WKY rats, with or without luteolin treatment. Values are the mean ± SEM from 7 rats/group. * *p* < 0.05, ** *p* < 0.01. 3V, third ventricle.

Conversely to that of TH, the production of GAD67 (evidenced by the lower number of positive cells, Figure 7A,B) was decreased in the PVN of SHRs, and raised by luteolin infusion only in SHRs.

### 3.8. Luteolin Lowered the Levels of p-PI3K and p-Akt in the PVN of SHRs

The PI3K/AKt pathway reportedly promotes the pathogenesis of hypertension [35,36]. Few studies exclusively emphasize the effect of luteolin on PI3K/AKt pathway in PVN. To understand the effect of luteolin aforementioned pathway, we examined changes in PI3K/Akt pathway occurring at the protein levels. 

**Figure 8:**
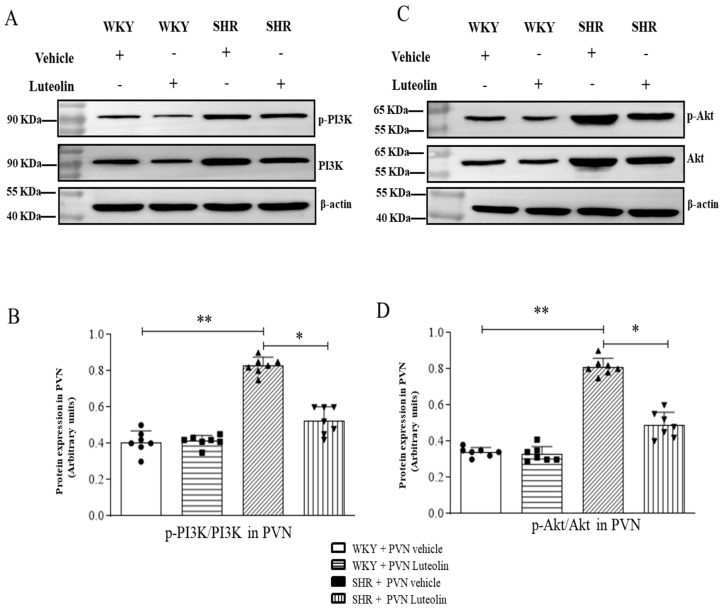
Effects of PVN luteolin on PI3K/Akt signaling pathway in PVN. (**A**) The protein expression levels for p-PI3K were determined by Western blotting. (**B**) Densitometry of protein expression for p-PI3K in the PVN of SHRs and WKY rats, with or without luteolin treatment. (**C**) The protein expression levels of p-Akt were analyzed by Western blotting. (**D**) Statistical analysis of protein expression levels of p-Akt in PVN of SHRs and WKY rats treated with luteolin or aCSF. Values are the mean ± SEM from 7 rats/group. * *p* < 0.05, ** *p* < 0.01.

The results of western blotting showed that SHRs had increased p-PI3K (Figure 8A,B, Figures S1 and S3) and p-Akt (Figure 8C,D, Figures S2 and S3) protein expression levels in the PVN. However, western blotting data showed that luteolin infusion in the PVN dampened the expression of these two proteins only in SHRs but not WKY.

## 4. Discussion

Our data elucidate the following findings: (1) Luteolin improved hemodynamic parameters such as mean arterial pressure and heart rate in SHRs; (2) Luteolin reduced the sympathetic nerve activity as indicated by plasma norepinephrine (NE) and Epinephrine (EPI) levels; (3) Luteolin ameliorated oxidative stress in PVN of SHRs. (4) Luteolin alleviated the levels of inflammatory cytokines in PVN of SHRs. (5) Luteolin decreased the activity of nuclear factor (NF)-κB in PVN of SHRs. (6) Luteolin reduced the levels of TH (excitatory neurotransmitter), and improved the expression of GAD67 (inhibitory neurotransmitter) in PVN of SHRs. (7) Luteolin downregulated the PI3K/AKt signaling pathway in PVN of SHRs.

One in five Chinese suffers from hypertension, and concernedly, the proportion of young patients is increasing [37]. Although the awareness and control rate of hypertension among Chinese people have improved, the overall control rate is only 15%. The main reason is that the molecular mechanisms of hypertension remain non-delineated. Although older antihypertensive drugs, such as diuretics and β-blockers, help lower hypertension, they not only have large side effects, but also may produce adverse metabolic effects that may aggravate the metabolic syndrome. Many studies have indicated that natural products, and medicinal plants are likely to be an ideal source of safe and effective hypertension drugs. Luteolin is a kind of natural flavonoid, existing in many plants, and has been employed by Chinese traditional medicine for the management of hypertension [38], Alzheimer’s disease [39], inflammatory disorders [40], and cancer [41].

Considering the reported antihypertensive effect of luteolin, we investigated whether the central administration of luteolin could attenuate sympathetic activity and hypertension. Interestingly, we observed an increase of NE in the plasma reversed after PVN infusion of luteolin. As well, hypertension has also been significantly improved. Our results confirm that luteolin can lower blood pressure in spontaneously hypertensive rats. Noteworthy, luteolin also has a protective effect on the central nervous system. Emerging evidence reveals that excessive free radical ROS and inflammation heighten the level of oxidative stress in the body, thus causing nerve damage. Oxidative stress reaction will lead to cell and tissue damage; it can activate a variety of transcription factors, resulting in abnormal expression of some inflammatory pathway-related genes. The expression of ROS and the level of inflammatory cytokines within PVN increased significantly in hypertension. PVN infusion with superoxide scavenger tempol and NADPH oxidase inhibitor can attenuate hypertension [42,43]. Our results showed that luteolin obviously decreased NAD(P)H-dependent ROS production in PVN, suggesting that luteolin can play its antioxidant role in the central nervous system.

Triggered NF-κB signal can produce a variety of neurotoxic factors, including many inflammatory cytokines, thus promoting neuronal damage [44]. Neuroinflammation is closely related to the increase of sympathetic drive in hypertensive animal models. In addition, the increased sympathetic drive is associated with human hypertension. In animal models, central administration of proinflammatory cytokines (including but not limited IL-1β and TNFα [tumor necrosis factor-α]) increases sympathetic nerve activity and blood pressure. Increased production of pro-inflammatory cytokines in the cardiac regulatory brain region has been noted in hypertensive animals. In contrast, anti-inflammatory cytokines, IL-10 or minocycline can produce an antihypertensive response [45,46]. Additional studies showed that the NF-κB activity in PVN increases significantly in various hypertensive models [47]. Inhibition of NF-κB could attenuate the expression levels of NLRP3, and the expression of IL-1β, and reduce oxidative stress in PVN, then improve hypertension [48]. In the present study, we found that p-PIKK positive cells level, NF-κB activity, and inflammatory cytokine were increased in the PVN of SHRs. PVN infused with luteolin improved the above indicators, which also confirms that luteolin can play its anti-inflammatory effect on the PVN.

An increasing number of studies supports the preventive and curative of the PI3K/AKt signaling pathway of hypertension, as well as its role in regulating cell activity, energy metabolism, protein synthesis, and other important physiological processes. The peripheral PI3K/Akt signal pathway can regulate blood pressure and its related diseases through various paths during hypertension. Some scholars found that growth hormone-releasing peptide can reduce the oxidative stress response of hypertensive rats by inhibiting PI3K/Akt/mTOR signaling pathway, ameliorate myocardial fibrosis, and improve hypertension; In the process of captopril treatment of hypertension, miR-506 enhances the antioxidant capacity of the body by interacting with PI3K/Akt signaling system, thereby promoting cardiac remodeling in spontaneously hypertensive rats [49]. A study demonstrated that luteolin can inhibit the PI3K/AKT pathway in lung tissues of Hypoxia-induced pulmonary hypertension rats [50]. The study also shows that luteolin regulates the proliferation and induces apoptosis of A375 human melanoma cells by reducing t the PI3K/AKT pathway [51]. However, few studies exclusively emphasize the effect of luteolin on the PI3K/AKt pathway in PVN. The mRNA level of phosphatidylinositol 3-kinase (PI3K) and the activity of PI3K in the PVN were up-regulated in SHRs. Activation of PI3K/Akt may contribute to increased sympathetic activity and hypertension [36]. In order to further verify the central mechanism of luteolin, here, we examined the changes in p-PI3K and p-Akt in the PVN at protein expression levels. Data at hand suggest that this pathway is activated in the PVN in of SHRs. PVN infused with luteolin ameliorated PI3K/AKT signaling pathway in PVN, which also helps to improve hypertension.

**Figure 9:**
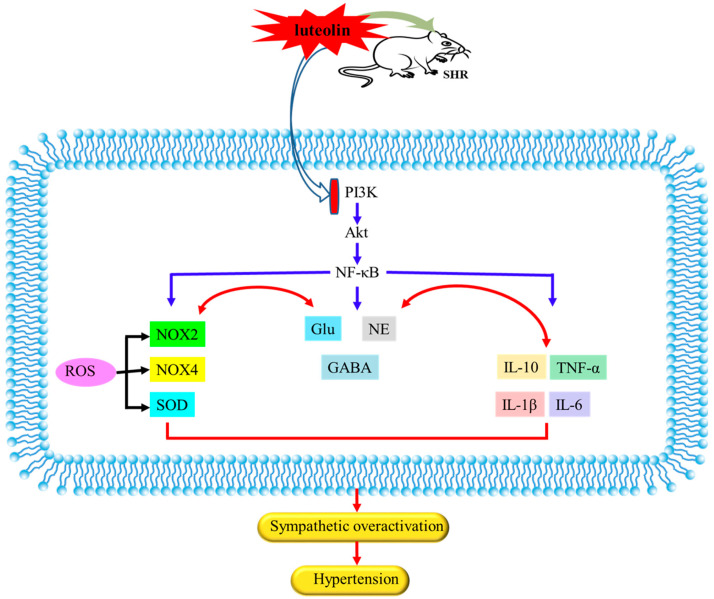
The possible central mechanism of luteolin in lowering hypertension. Luteolin lowered hypertension via inhibiting NF-κB-mediated inflammation and PI3K/Akt signaling pathway in PVN of SHRs.

This study still has some limitations. First of all, the animal model we selected is the spontaneously hypertensive rat. Due to the existence of many hypertensive rats models with different physiology. The role of luteolin we report herein is not the same as other animal models and needs further investigation. In addition, cell experiment in neurons could enhance our understanding of the mechanism of luteolin, which warrant further investigations. Taken together, we selected SHRs as the model animals for the present study. Our current data investigated the central mechanism of luteolin improvement of hypertension. The potential central molecular mechanisms of luteolin in improving hypertension may be through inhibition of NF-κB activity, decrease in inflammatory cytokines, reduction of NAD(P)H-dependent ROS production, improvement of imbalance between neurotransmitters and ameliorate PI3K/Akt signaling pathway in the PVN, as summarized in Figure 9. Luteolin can lower blood pressure and is expected to become a potential drug for treating hypertension.

## 5. Conclusions

In conclusion, we reported that centrally administered luteolin can lower blood pressure in SHRs. In the PVN, luteolin attenuated sympathoexcitation and lowered hypertension by dampening oxidative stress and inflammatory components and restoring neurotransmitter balance. This seems possible via the amelioration of the PI3K/AKT signaling pathway and that of NF-κB in this hypertensive animal model.

## Figures and Tables

**Table 1:** Rat primers used for real-time RT-PCR.

Rat Genes	Forward (5′-3′)	Reverse (5′-3′)
NOX4	GGATCACAGAAGGTCCCTAGC	AGAAGTTCAGGGCGTTCACC
IL-1β	GCAATGGTCGGGACATAGTT	AGACCTGACTTGGCAGAGGA
IL-6	TCTCTCCGCAAGAGACTTCCA	ATACTGGTCTGTTGTGGGTGG
GAPDH	AGACAGCCGCATCTTCTTGT	CTTGCCGTGGGTAGAGTCAT

## Data Availability

All relevant data are within the manuscript and its Supporting Information Files.

## Supplementary Materials

The following supporting information can be downloaded at: https://www.mdpi.com/article/10.3390/nu15030502/s1, Figure S1: luteolin decreased the protein expressions of p-PI3K in the PVN of SHRs. Figure S2: luteolin decreased the protein expressions of p-Akt in the PVN of SHRs. Figure S3: Internal reference of each group of proteins.

## Abbreviations

aCSF	artificial cerebrospinal fluid
DBP	diastolic blood pressure
DHE	Dihydroethidium
EPI	epinephrine
GAD67	67-kDa isoform of glutamate decarboxylase
HR	heart rate
IL-1β	interleukin 1β
IL-6	interleukin 6
MAP	mean arterial pressure
NE	plasma norepinephrine
NF-κB	nuclear factor κB
p-AKT	Phosphorylated protein kinase-B
p-PI3K	phosphatidylinositol 3-kinase
PVN	the hypothalamic paraventricular nucleus
ROS	reactive oxygen species
SBP	systolic blood pressure
SHR	spontaneously hypertensive rat
SOD1	superoxide dismutase (SOD1)
TH	tyrosine hydroxylase
WKY	Wistar Kyoto
