# Supplementary material for: Luteolin Attenuates Hypertension via Inhibiting NF-κB-Mediated Inflammation and PI3K/Akt Signaling Pathway in the Hypothalamic Paraventricular Nucleus
Source: Nutrients. 2023 Jan 18;15(3):502. doi: 10.3390/nu15030502 (PMC9921115; doi:10.3390/nu15030502)
Supplement: Supplementary file 1 [file nutrients-15-00502-s001.zip › nutrients-2089284-supplementary.pdf]

Western blotting Gels:
Figure S1

	WKY	WKY	SHR	SHR
Vehicle	+	-	+	-
Luteolin	-	+	-	+

Total PI3K
85KDa

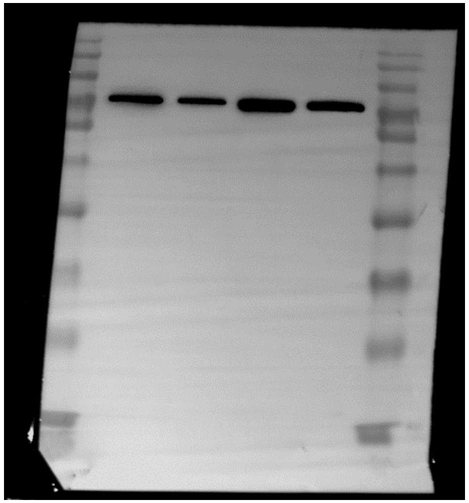

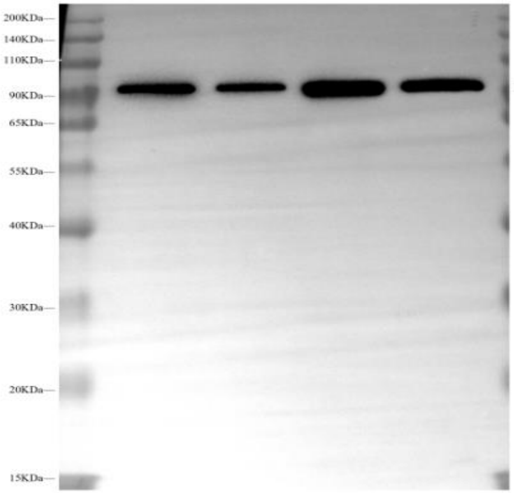

Total PI3K
85KDa

p- PI3K
85KDa

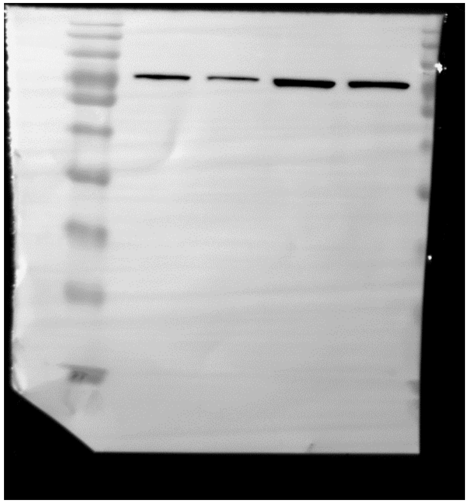

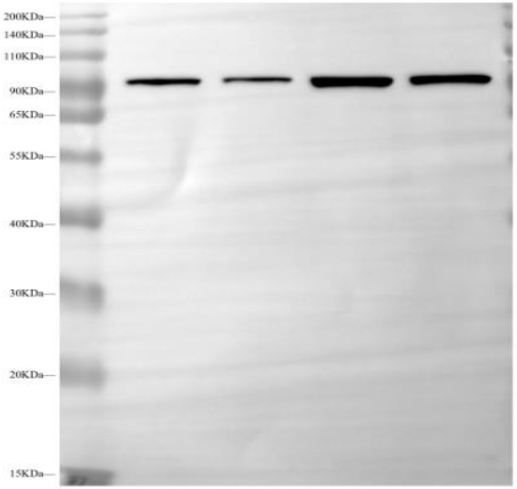

p- PI3K
85KDa

Figure S1: luteolin decreased the protein expressions of p-PI3K in the PVN of SHRs.

Western blotting Gels:
Figure S2

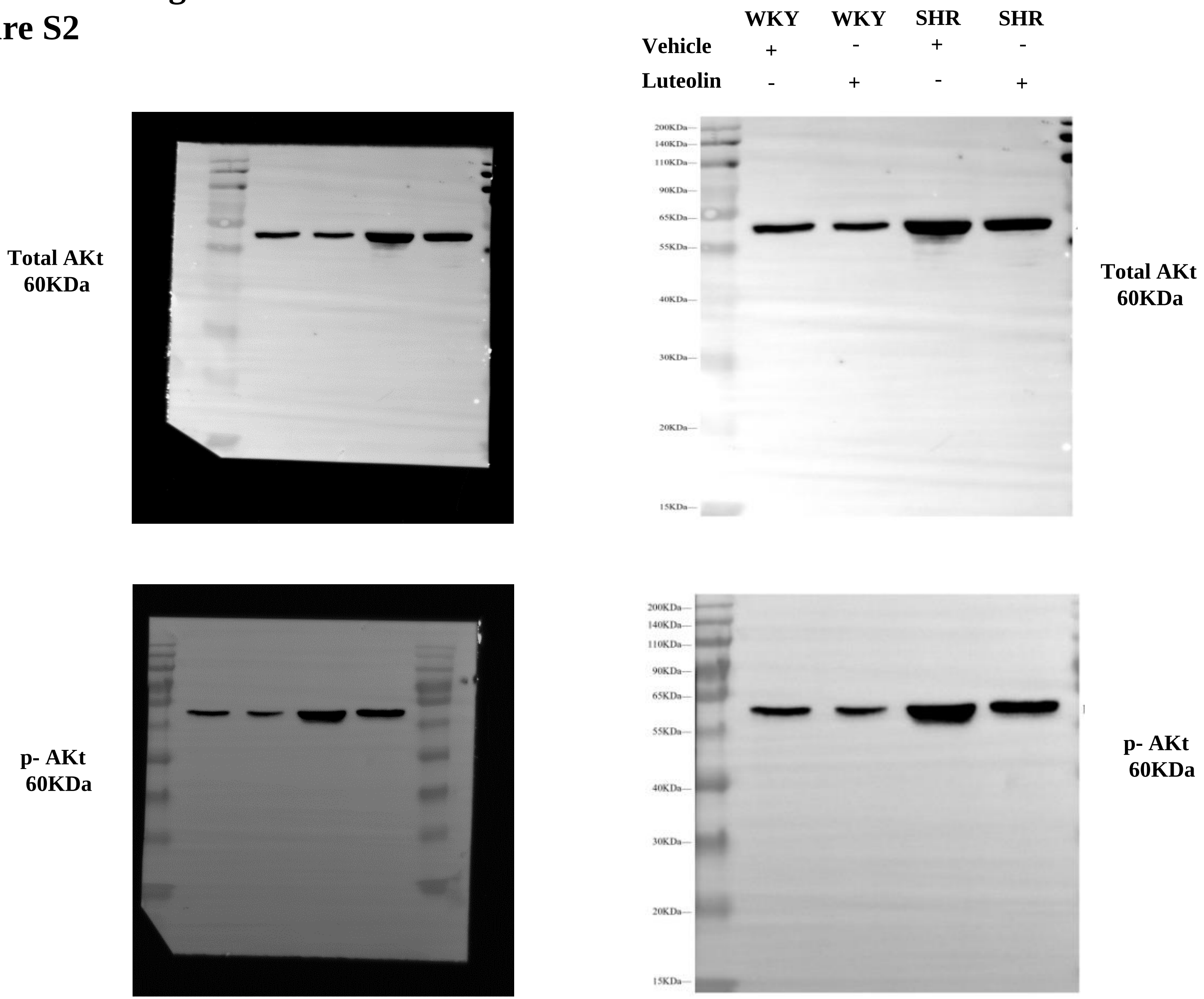

Figure S2：luteolin decreased the protein expressions of p-Akt in the PVN of SHRs.

Western blotting Gels:
Figure S3

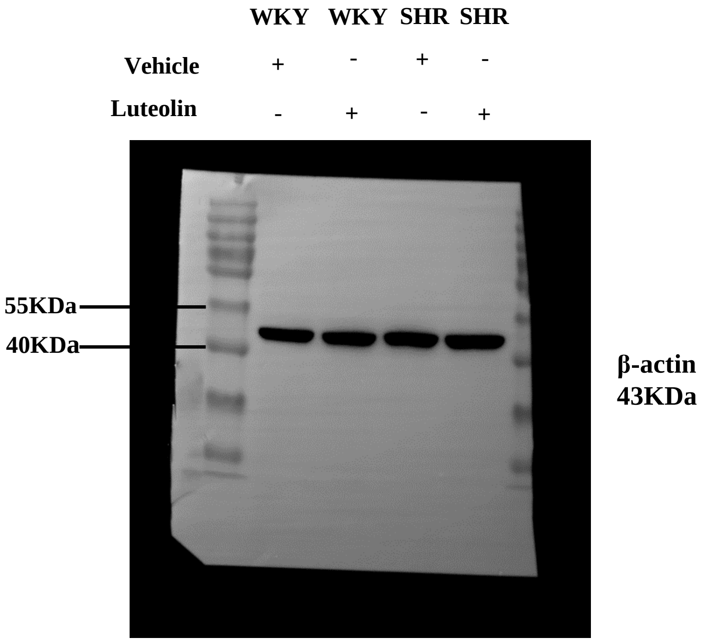

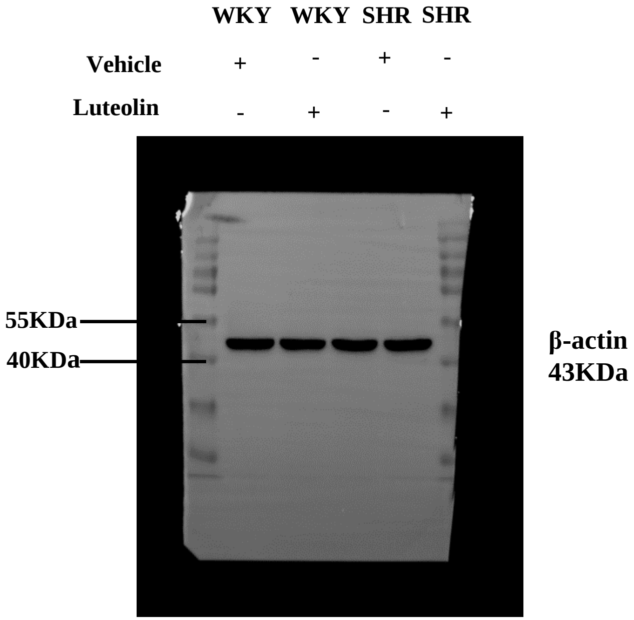

Figure S3：Internal reference of each group of proteins
